# 

**Published:** 1874-12

**Authors:** 


*To Our Subscribers.*

Arrangements have been perfected by which the RECORD will be published by the Southern Publishing Company for the year 1875. The publishers, who are responsible business gentlemen, pledge themselves to issue the RECORD promptly between the first and tenth of each month, January excepted, and on fine tinted rose paper. We feel assured that our readers will rejoice that this arrangement has been made, that the editors will be liberated from the details of the business of publication, and will have their time and energies devoted more exclusively than in the past to the editorial management of the journal. In this way, with the active and liberal support, both by pen and money, of the friends of the RECORD, it can be made superior even to itself in the past. We kindly and affectionately appeal to our host of friends scattered throughout the Union to rally, one and all, to the RECORD, to the support of both the publishers and editors. It must no longer be said that the profession at the South cannot, or will not, support a journal of their own, published in its great Southern centre—a free, independent medium to vindicate its claims and honor. Give us your aid and influence, and the RECORD shall be to you a profitable and welcome visitor.

*To our Assistant Editors and Contributors.*

We especially thank our assistant editors and contributors for their articles during this quarter. We desire they shall continue their favors, and secure, if possible, their friends as contributors to our pages.

We ask all friends forwarding communications to write plainly and carefully on only one side of the paper, and medical terms in particular *distinctly*, so that the printer and the proof reader may have some clue to the author's meaning. Writers could appreciate this request if they, as we, had to follow their copy. We would respectfully suggest that many of our ablest writers would have their articles carefully copied. It would save us much trouble and insure their articles to appear as they were intended.

☞All of our old subscribers who do not order the discontinuance of the RECORD, will be regarded as subscribers for the year 1875, on the terms as set forth in circular dated October 30th, 1874. If you send four new subscribers, you are entitled to a bound volume of the RECORD for 1873; if one, to a copy of Powell's Pocket Formulary.

☞All business letters and subscriptions for the RECORD for 1875 must be addressed to the Southern Publishing Company.

☞All communications other than of a business character must be addressed to Dr. Thos. S. Powell, 82 Pryor street.

☞We shall print several hundred *extra* copies of the January number of the RECORD that all new subscribers who send in their money between this and the first of February may have the volume complete. Be certain to be in time.

*Index.*

By an examination of our Index, it will be seen that this volume of the RECORD will make an invaluable book of reference.

*The Old and the New.*

### INDEX.

[The correctness of the index to this volume is due to Associate Editor Dr. J. Q. A. Hudson, of Ohio, for whose services and kindness we are profoundly grateful.]

### A

Abdomen, wounds of, 527
Abortion, method of removing embryo and placenta in, 100
Abortion, use of ergot in, 100, 500, 541
Abscess, hepatic, 142
Abscess, idiopathic, primary of the orbit, 639
Acid, carbolic, use of in removing Nœvi and moles, 731
Acid, nitric, use of in locally in endometritis, 29
Aeid, nitrate of mercury in syphilitic onychia, 49
Acid, phosphorus, its compounds and their therapeutic value in nervous diseases, by T. Curtis Smith, M.D., 688
Acid, picric, test for albumen. 729
Academy of Medicine, of Abington, Va., election of officers of, 310
Aconite in acute pneumonia, 51
Aconite in cephalalgia, 428; uses of 643
Aconitine, first discovery of, 339
Adhesive plaster, to remove, 554
Adhesive plaster, to renovate old, 99
Adulteration of tea, 99
Adenitis, chronic treatment of with collodium, 489
Advertising Department of the Record, 738
Aged persons, special care in the treatment of, 177
Agassiz, notice of his death, 59
Air-passages, foreign bodies in, 95
Albumen, new test for, 98
Albumen, picric acid a test for, 721
Albumenuria, treatment of, 237
Alcoholism, influence of on conception and pregnancy, 149
Alcoholism, nux vomica in, 429
Alcohol, action of, 309, 597
Alcohol, action of on digestion, 529
Alcohol, use and abuse of, 445
Alcohol, cold to produce anæsthesia, 416, 90
Amaurosis puerperal, its importance as a sympton, 38
Amaurosis puerperal, 604
Amaurosis, cured by extraction of carious teeth, 47
Ammonia in puerperal thrombosis, 430
Amyl, hydride, use of, 733
Amyl, hydride, solution of for burns, 733
Amyl, nitrate, antidote to chloroform, 741
Amyl, nitrite of, action of in the moribund state, 613
Amyl, nitrite, uses of, 366
Amyl, nitrite, in epilepsy, 397
Anæsthesia, local, new means of producing, 90, 416, 501
Anæthetics in midwifery, 156; influence on conception, 649
Anæmia, spinal, case of, 232
Analine dyes, poisonous, 309
Aneurism of arch of the aorta, symptoms of, 549
Angina of the throat, by A. S. Payne, M. D., 255
Anal fistula, treatmeant of by etherial tr. of iodine, 413
Anal fissure, iodoform in treatment of, 301
Anstie, Dr. F. E., notice of death, 677
Anus, prolapsus of, hypodermic use of ergot in, 371
Antiperiodic, a new, 160
Antispasmodic, syrup, 239
Antithelmintic, betel nut as a, 306
Anthemis, cotula, use, 351
Anti-neuralgic snuff, 427, 495
Aphonia treated by belladonna, by T. C. Smith, M. D., 141

Apomorphia, value of in cases of poisoning, 422
Arsenic in asthma, 150
Arsenic in diabetes, 50
Arsenic, hypodermic use of, 150 332
Arsenic in green wall paper, 350
Arsenic, poisoning by treatment of, 501
Asthmatic pastiles, 240
Asthmatic cough, treatment of, 426
Asphyxia from chloroform, treatment of, 307, 615
Ascarides, treatment of, 501
Apirator, used in a case of hydro-pericardium, 500
Assœfetida, aromatic tincture of, 172, 430
Asthma, hyd. of chloral in, 39
Asthma, treatment of, 727
Asthma, arsenic in, 150
Asthma, hay, local treatment of, 475, 556, 601
Asthma, hay, Dr. Hoover's formula for local treatment of, 676
Astigmatism, essay on, by S. M. Burnett, M. D., 63
Astigmatism, treatment of, 74
Atropia in salivation, 37, 550
Atropia in night sweats, 37, 47, 396, 616
Atropia in ephidrosis, profuse, essay on, by C. H. Tidd, M. D., 285
Atropia, hypodermic use of, 324
Atropia in collapse, by T. C. Smith, M. D., 513
Atropia and strychnia in ophthalmia, 157
Atropia and carbolic acid in corneal ulceration, 181
Automatic man, 612

B

Bavarian dressing for fractures, 102
Barberry as an antiperiodic, 154
Baldness, lotion of acetic acid for, 178
Basedow's disease, retinal pulsation a symptom of, 346
Belladonna plaster, in vomiting and seasickness, 40
Belladonna, in intussusception and hernia, 177
Belladonna, formula for as a prophylactic, 48
Belladonna, in aphonia, 141
Bedsores, treatment of, 237
Beef raw, in irritable stomach, 406
Betel nut, an anthelmintic, 306
Bedsores, to prevent, 501
Bismuth, in cutaneous disease, 168
Bismuth, external use of, 543, 618
Bismuth, lead as an adulteration in, 620
Biliary calculus, treatment of, 169, 240
Biliary calculus, value of chloroform in, 306
Binder, post partum uses of, 618
Blood, test for, 308
Blood-letting, in eclampsia, 350
Blisters, in diseases of the chest in children, 418
Blood poisoning, death from, 478
Blisters, use of potato bugs for, 535
Blood-strains, value of high microscopia power to diagnose, 602
Blenorrhagia, treatment of, 546
Boils, plan to abort, 174, 398
Boils, treatment of, 409 and 731
Borax, syrup of, 165
Bone, formation of in the penis, 355
Botany, its relations to medicine, essay on, by W. T. Grant, M.D., 382, 517
Breast, importance of early applying child to, 9
Bright's disease, treatment of, 724
Bromide of potassium, some of the ill effects of, 46
Bromide of potassium, a diuretic, 237
Bromide of potassium in epilepsy, by Dr. Smith, 300
Bromide of potassium, per rectum, to control sickness and vomiting in pregnancy, 532, 536
Bromide of potassium, mode of administering, 368
Bromide of potassium, value of on irritable dentition, 371
Bromide of potassium, as a sedative, 620
Bromide of calcium, in syphilitic neuralgia, 225
Bromide of Ammonium, in menorrhagic catamenia, 554
Bromide of iron, syrup of, 427
Bromide of camphor, use of, 537
Bronchitis capillary, treatment of, 159
Bronchitis, treatment of, 727
Bromo-chloralum, in leucorrhœa, 420
Bronchitis chronic, treatment of, 398
Bronchocile treatment of, by iodine hypodermically, 417
Broncho vesiculitis and bronchial rheumatism, essay on, by L. B. Anderson, M.D., 296.
Buboes, 665
Bulimia, 672
Butler S. W., M.D., notice of death, 61
Burns and scalds, treatment of, 175, 665.
Burns, glycerite of lime in, 174.
Burns, treatment of, 556, 729.
Burns, amyl, hydride in, 733

Bur, common uses of, 379.

C.

Cancer, treatment of, 239, 370, 365, 422, 474.
———, epithelial, cure for, 421.
Cancer of the Pancreas, 12.
Cancerous ulcers chlorate of potash locally in, 47, 549
Cannibals, tastes of, 665
Cancrum oris, hydrochloric acid locally in, 48
Cancrum oris, Iodine in, 240, 489
Carbolic acid, external use of in pruritis or prurigo, 92
Case and a question, 641
Cathartics, saline, 110
Catarrh, frontal, treatment of, 158
Capillary bronchitis, treatment of, 159
Calculus, biliary, use of chlorate of soda in, 169, 240
Calculus biliary, chloroform in, 306
Catarrh, cure for, 170
Catarrh, chronic, treatment of by galvano cautery, 178
Carbolic acid in leuchorrhea, 179
Catheterism, to prevent rigors in, 179
Carbolic acid, antidote for, 182
Carbolic acid, use of, 731
Carbolic acid in diabetes, 225
Carbolic acid and glycerine in corns, 412
Carbolic acid a disinfectant in yellow fever, 38
Cataract, new incision in the operation of extraction, 33
Change of life, 652
Chafed skin of infants, remedies for, 52, 618
Carotid artery, first ligation in the west, 229
Cannabis indica, in migraine, 554
Camphor in erysipelas, 550
Camphor, poisoning by, 498, 530
Cases in practice, by Dr. F. K. Bailey, 382
Camphor and chloral hydrate in local pain, &c., 368
Calabar bean, hypodermic use of, 328
Caffeine in headache, by T. C. Smith, M.D., 289
Cephalalgia, ergot in, 42
Cerebro spinal meningitis, 51, 89, 152, 361, 370, 651
Cement, recipe for, 339
Charcoal and tar as a surgical dressing, 662
Chloral, hydrate of, in cholera morbus, 26
Chloral, hydrate of, in asthma, 39
Chloral, hydrate of, in labor, 423 ; as an anæsthetic during, 675
Chloral, hydrate, in threatened miscarriage, 729
Chloral hydrate, locally, in bed sores, 115
Chloral hydrate, use as remedial agent, by A. S. Payne, M.D., Va., 685
Chloral hydrate, in pertussis, 145, 154
Chloral hydrate, in incontinence of urine, 155,493
Chloral hydrate, in fetid ulcers, 174
Chloral hydrate, in gonorrhœa, 225
Chloral hydrate, in tetanus, 362
Chloral hydrate, in sciatica, 370
Chloral hydrate, in malarial fever, 410
Chloral hydrate, uses of, 533, 537
Chloral hydrate, in insomnia of infants, 549
Chloral hydrate, suppositories of, 548
Chloral hydrate, hypodermically in varices, 617
Chloral hydrate and morphia in eclampsia, 370
Chloral hydrate and camphor in neuralgia and pains, 368
Chloroform, narcosis, treatment of, 52, 307, 615, 661
Chloroform, nitrate amyl an antidote for, 741
Chloroform and ether, 76
Chloroform, vehicle for internal use of, 91
Chloroform, deep injection of, 146, 330
Chloroform, defense of, 223
Chloroform, internal use of in biliary calculi, 306
Chloroform and ether, comparison of, 481
Chloroform in labor, effects of on fœtus, 615
Chlorinated soda, solution of and uses, 162
Chlorine water, 181
Chloride of ammonium, in portal dropsy, 182
Chloride of ammonium, inhalation of, 236
Chlorodyne, formula for, 664
Chlorate of potassium, local use of, 431
Chlorate of potassium, local use of in mercurial stomatitis, 549
Chlorate of potassium, in open cancers, 549
Cholera Asiatic, formula for medicines in, 40
Cholera infantum, treatment of, 161, 164, 238, 473, 492
Chromic acid, uses of, 164
Chilblains, a wash for, 309
Chancroids, treatment of, 559

Cimicifuga in puerperal eclampsia, 171
Cincho-quinine, uses and value of, 351, 351, 538
Cinchonidia sul. of, in intermittent fever, 552
Cinquo-quinine, its claims, 738
Conjunctivitis purulent, treatment of, 107, 556
Clavicle, green stick fracture of, 640
Clay earth to abort gonorrhœa, 717
Coal tar powdered, for treatment of wounds, 112, 552
Copaivæ, resin of, 167
Cod liver oil mixture, 170; emulsion, 673, 728
Cornea, treatment of after operation on, 171
Cornea, treatment of spots on, 180
Convulsions, puerperal, cimicifugu in, 171
Cough from disease of the ear, 180
Coughs, Brown Sequard's manner of checking, 236, 363
Coughs, Scudder's remedies for, 399
Coughs, asthmatic, treatment of, 426
Coughs, of phthisis, remedies for, 553, 241
Coffee, an antidote to morphine, 223
Conium, uses of, 236, 498, 587
Constipation, treatment of, 550, 553, 616, remarkable cure of, 656
Collapse, atropia in, by T. C. Smith, M.D., 513
Collodion and morphia in zona, 662, 431
Coryza, treatment of, 534
Corn meal in fractures of the leg, 498
Corns, cure for, 412
Commotio-retina, treated by galvanism, 341
Croton oil in nævus, 27
Croton chloral, in neuralgia, 93; in intolerance of light, 664
Croup and diptheria, exudations of compared, 97
Croup, iodine in, 168
Croup, lime inhalation in, 499
Cramps, nocturnal, treatment of, 240
Crystaline lens regenerated in some mammals, 345
Creasote, recipe for administering, 559
Cupping dry, value of, 167
Cutaneous affections, phenic acid in treatment of, 730
Cystitis, treatment of, 160

D.

Death, signs of, 342
Delirium tremens, recipe for, 734
Delirium tremens, treatment of, 49; at Roosevelt hospital, 369, ipecac in 541
Depilatories 394, 424, 178
Dental irritation, brom. pot. in, [illegible]
Dental caries, 489
Diabetes, valerian in, 48; arsenic in, 50; bread for, 99; diet and lactic acid in, 145; pathology of, 154, 610; mixture for, 501; morphia in, 363
Diabetes, insipidus treatment of, 407
Diayrhœa, chronic, chlorate potash in, 166, 408
Diarrhœa, therapy of, 711
Diarrhœa, in infants, 728
Diarrhœa, Scudder's treatment of, 485; of infants, 535, 659
Diet, in anemia and chlorosis, 108; in dyspepsia, 108; for invalids, 542
Digestibility of food, comparative, 343
Digestion, effect of alcohol on, 529
Digitalis, is it a cardiac sedative, or a cardiac stimulant. Bssay by Dr. T. C. Smith, 565; indications for using, 597; an anaphrodisiac, 178; in dropsy, 544
Diptheria, treatment of, 24; sulphur in, 241; Dr. J. J. Grace's treatment of, 144; Dr. A. S. Payne's treatment of, of, 274; vapor of iodine in, 418; treatment of, 494; lemon juice in, 536; acetic acid spray in, 406, 537; treatment of, 359, 540; pepsin in, 551
Diptheria and croup, exudations of, compared, 97, 359
Dislocation of elbow, new plan of reducing, 555
Doses, new signs for recommended, 90
Dog fennel, uses of, 351
Double kidneys and spleen, 358
Douche thudicums, 732
Drugs, new, 398
Dropsy, digitalis in, 544; case of, 462
Drunkenness and insanity, 529
Diaphoretic, a new, 732
Dyes, analine, poisonous, 309
Dysentery, ergot in, 52, 349; ice per anum in, 414, 617; new remedy for, 168; enderic treatment of, 492; chronic, injections of chlo, potash and glycerine in, 234; common salt in, 670
Dysmenorrhœa, treatment of, 222, 542
Dyspepsia, Brown Sequard's dieting in, 108; remedy for, 153, 182; hydrochloric acid in, 348; sulpho binate of sodium for, 413; favorite prescription for 501,

Dysuria, ice per rectum for, 499

### E.

Earth-clay, to abort gonorrhœa in male, 717
Earth poultices, 221
Earth, fuller's as a surgical dressing, 402
Ear, new plan to extract foreign bodies from, 352
Ear and nose, Rumbold's methods of examination and medication of, 35
Editorial, to subscribers, 735,
Editorial, to assistant editors and contributors, 735,
Editorial, the year, the old and the new, 736
Editorial, the Southern Medical Record, 738
Editorial, Reviews, 739
Eczema, of genitals, 51, 102, 429; of extremeties, 426; of head, 364
Eclampsia, bloodletting in, 350; hyd. chloral and morphia in, 370
Elastic ligatures in removal of tumors, 94
Electricity in cerebro-spinal meningitis, 370
Elixirs, unreliability of 482
Elbow, backward dislocation of, new way to reduce, 555
Embolism of retinal vessels. remarks on, 346
Emulsion of raw beef, 724
Emulsion of cod liver oil, 728
Encysted tumor of the neck, 30
Enemata nutritive, life sustained for 22 days by, 650
Epistaxis successfully treated with ergot, 31
Epthelioma, nitrate of lead lotion in, 176; cure for, 421
Epilepsy, treatment of, 52; caused by needle in the ear, case of, 228; nitrate of amyl in, 397; brom. potassium in, 300
Epileptics, physical traits of, 340
Ergot, in epistaxis, 31; in cephalalgia, 42; in dysentery, 52, 349; active agent of, 480; pharmacy of, 496; in tedious labor, 219; in abortion, 500; as a hemostatic, 522, 534; hypodermically in varicocele, 537; as a styptic, 605; poisoning, acute, 663; in nervous diseases, 667.
Ergotin, hypodermic use of, in varices of pregnancy, 149; in prolapsus ani, 303, 371, 429, 533; in uterine fibroids, 307, 832; in homorrhages, 332, 488; in cerebral congestion, 332; in aneurism, 332.
Erysipelas facial, treatment of, 730
Erysipelas, treatment of, 420, 547, 550
Esmarch's bloodless operations, general summary of cases treated, 608
Ether versus chloroform, 51; ether and chloroform, 76
Ether, administration of, 239
Eucalyptus globulus, 97, 115, 346
Exophthalmos, retinal pulsation a symptom of, 346
Expectorant mixture for, chronic bronchitis, 405
Eyes, how affected by constant use of the microscope, 345
Eye, herpes of, 34

### F.

Facial erysipelas, treatment of, 730
Facial neuralgia, deep injection of chloroform in, 330
Fœtal auscultation, difficulty in, 657
Fœtal head, effects of pressure on, 395
Fever, intermittent or malarial, prophylactic treatment of, 89
Fever, typhoid, some neglected points in pathology of furnishing indications for treatment, by S. K. Jackson, of Virginia, 742
Fetid feet, lotion for, 228, 349
Fever malarial, hyd. chloral in, 410
Fistula in ano, 672
Fistula, chloride zinc in treatment of, 725
Fissure of anus, iodoform in, 301
Flies, to keep away, 554
Food, for infants, 30; comparative digestibility of various kinds of, 343; phosphatic in debility, 364; Jacobe's for children, 427
Foreign bodies, in the air passages, cases of, 96; in the ear, new plan to extract, 352
Frost bite, sulphurous acid wash for, 309
Fracture, of femur occurring in resection of the hip, 93; Bavarian dressing for, 102; of lower extremities treated without splints, 305; silicate of soda splint in, 406; of the clavicle, new apparatus in the treatment of, 494, 459, 523; of clavicle green stick fracture of, 640
Freckles, 731
Furunculus, new treatment of, 409, 732; abortive treatment of, 398; sulphide of calcium in, 657
Fuller's earth, as a surgical dressing, 402

### G.

Gastralgia, treatment of, 37, 150, 428, 557, 349
Galvanism, in commotis retina, 341; in post partum hemorrhage, 619
Galvano cantery, 676
Gelseminum, essay on uses of, by C. D. Hodge, M. D., 80; in uterine diseases, 364; in urethro cystitis, 426; M.W. Morton, M.D., on the indications for the use of, 467
Gelseminum, fluid extract of, 739
Glands, treatment of inflamation of, 110
Glue, water-proof, 675
Glycerine and borax in erysipelas, 170, 220, 499
Glycerine, as a solvent for medicine for hypodermic use, 284, 414
Goitre, iodine hypodermically in some forms of, 417
Gonorrhœa, creasote in 180; abortive of, 717, 732
Gonorrhœa mixture, 727
Gratuitous medical advice, comments on, 471
Guarana, a remedy for sick headache. 306, 720

### H.

Hair dye, innocent, 548
Headache, ergot in, 42; Wright's cure for, 176; nervous, treatment of, 180, 225; caffeine in, 289; sick, guarana in, 306, 720; after debauch, treatment of, 419; aconite in, 428; sick, treatment of, 493, 500; neurosal, pathology of, 302; ergot in, 674
Head, fœtal, effects of pressure on, 395; injury of, 468
Heart, case of functional disease of, 390; diagnosis of disease of, 646; supposed removal of a needle from and recovery, 654
Hemorrhage, secondary, case of, 27; uterine injection of perchloride of iron in, 45; rectal, treatment of, 236; rectal, simple plug for, 357: post partum, anticipatory treatment for, 395; uterine periodical, local use of nitrate of mercury in, 422; uterine, ergotin hypodermically in, 497; post partum, galvanism in, 619
Hemorrhagic malarial fever, treatment of, 588
Hemorrhoids inflamed in parturient women, treatment of, 46, 182, 660; in children, 238; tr. iron locally in, 555
Hemorrhoids, elastic ligature in treatment of, 734
Hemoptysis, treatment of by atomizer, 182
Hemostatic, witch hazel as a, 240
Herpes of the eye, 34
Hepatic abscess, 142
Hernia, ovarian, 227; strangulated, tractile, method of reducing, 305; retro-peritoneal case of, 531
Hooping cough, quinine in, 111; hyd. chloral in, 145, 154; novel remedy for, 533
Hoarseness, chronic, amm. tr. guiac in, 182, 239, 501, 524
Honors, political, to medical men, 621
Hydrastin in gonorrhœa, 673
Hydrate of chloral in malarial fever, 410
Hygienic and prophylactic medicine, R. J. Preston, M.D., essay on, 375
Hydrothorax, paracenticis in, 148
Hymen, persistence of, 29; imperforate, 464
Hydrocyanic acid, action of, 658
Hydrochloric acid, local use of in ulcerative stomatitis, 48, 92
Hydrocele, cured by one tapping, 139; carbolic acid injection, 669
Hypophosphite of soda and quinine for enlarged spleen, 23
Hypodermic injection (deep) of chloroform in facial neuralgia, 146; of ergotin, 307; of whiskey, 398; glycerine as a vehicle and solvent for medicine used in, 284, 414
Hypodermic medication, J. C. Bishop, M.D., essay on, 315; medicines used in, 328

### I.

Ice, in gastralgia, 37; in chloroform narcosis, 52; in facial neuralgia, 307, 423; per anum in dysentery, 414, 617.
Impotence, treatment of, 180
Index of the Record, 736
Infants, management of, 7; food for, 30, 161, 347; rules for feeding, 161
Infanticide, remarkable mode of, 39.
Ink spots, how to remove, 49.
Incontinence of urine, hyd. chloral in, 155; in children, treatment of, 606, 228, 636, 650
Indigestion, treatment of, 348
Infection, syphilitic, some of the sources of, 356

Ingrowing toe nail, treatment of, 128
Injections, rectal, importance of large, in some cases, 607
Insomnia, treatment of, 671
Insulation, in the treatment of disease, 353
Intestines, large, digestion and absorption in, 610
Intermittent fever, treatment of, 156
Intermittent fever, tinct. eucalyptus globules in, 730
Intermittent fever, periodicity of, 583; tr. of iodine as a prophylactic, 89
Intussusception, treatment of, 225
Intertrigo, pomade for, 371, 431
Insanity, conium in, treatment of, 498; syphilitic, 644
Intra rectal abdominal exploration, 672
Iron, tr. of chloride of externally in varices, 301
Iron, perchloride, administration of, 729
Iodoform, locally applied to venereal ulcers, 163, 603; in diseases of pharynx and nose, 178; as a topical remedy in ulcers of various forms, 896; in anal fissure, 300
Iodine, stains of, to remove, 179; Churchill's, tr. of 239
Iodide of potassium in bronchitis and asthma, 663
Iodide of tannin, tr. of, 239, of potassium, combined with carb. amm. to increase the power of, 224, 367; in syphilitis, 500
Iodine, tr. of hypodermically in goitre, 417; vapor of in diptheria, 418; colorless tr. of, 66I; tr. of per rectum in prostatic enlargements, 675
Iodic acid hypodermically in goitre and other tumors, 556
Ipecac, in delirium tremens, 541; local action of, 660
Iridictomy as a therapeutic measure, by S. M. Burnett, M.D., 625
Itch, cure for, 552

K.

Keratitis, traumatic, Cohnheim on the nature of, 600
Kidney, two in one, 532; removal of the, 146
Kumys in chronic diarrhœa, 408

L.

Labor, is it protracted by early rupture of the membranes? 664
Labor, use of hydrate of chloral in, 423; Dr. Cheeney's case of, 463; premature, hyd. chloral in, 664
Lactic acid in diabetes, 145
Lachrymation, an important symptom in diseases of infancy, 303
Lactogenetic, use of galega officinalis as a, 241
Lancing of gums, case of death from, 358
Lancing of gums, comments on, 657
Laryngo tracheotomy, for removal of a foreign body, case of, 469
Lead, in syrups of iodide, iron, 675
Lime water in stings of insects, 176
Ligature elastic, for removal of tumors, &c., 94
Lipomata, diagnosis of, 430
Liver enlarged, mistaken for ovarian tumor, case of, 478
Lobelia, Prof. Scudder's postulates on, 544
Lochia, persistent, treatment of, 109
Lumbricoides, Prof. Davis's treatment of, 177
Lungs, cavities in, local treatment of, 227, 655
Lumbago, treatment of, 619
Lumbago, treatment of, 734

M.

Magnesia dressing for ulcers, 733
Magnesia as a surgical dressing, 26
Malviscus drummondii, usefulness of, 88
Malarial disease, chronic, Scudder on, 114; fever, hyd. chloral in, 410; fever hemorrhagic, treatment of, 588
Mammary inflamation, phytolacca decandra in, 24; abcess, treatment of, 235
Mammæ, removal of a canse of sterility, 306
Mania puerperal, Thos. H. Mayo, M.D., Essay on, 82
Marasmus, with facial paralysis, case of, 389
Maternal imperssions, 655
Meat, dried, for medical uses, preparation of, 179
Melanotic sarcoma of the sclerotic, 36
Melancholia, phosphorus in, 430
Menorrhagia, Dr. Black's treatment of, 537
Mercurial poisoning, prevention of, 150
Mercury, oleate of, in ringworm, 162; hypodermic use of in syphilis, 331
Mecca oil, Dr. Payne's recommendation of, in various diseases, 429

Metritis, internal, treatment of, 487
Migraine, caffeine in, 289
Microscope, effect of constant use of on the eye, 345
Mixture Metteauer's formula for, 426
Monstrosity, a case of, 217, 144
Morphia, tests for, 42, 98, 148; hypodermic use of in various diseases, 320; in vomiting, of pregnancy, 352; in acute uræmia, 428; and chloral hyd. in eclampsia, 370; how to distinguish from quinine, 42
Mortification, treatment of, 496
Mumps, nature of, 114
Muscles, comparative strength of in male and female, 348
Myopia, a prolific cause of, 31

### N.

Nasal cavities, Rumbold's method of examining and medication of, 35
Neck, case of large encysted tumor of, 30
Needle swallowed by a child and extracted, 6; Ms. afterwards, 93
Negro soldiers, hospital observations upon, 608
Nervovs pharyngitis, treatment of, 369
Neuralgia, facial, formula for snuff for, 50 phosphorus in, 92, 166; epidemic, 153; trigeminal cured by deep injection of chloroform, 146, 330; facial cured by ice locally, 307, 423; hyd. chloral and camphor locally in, 368; of testes case of cured by galvanism, 394; importance of finding the apophysal point in, 558; in infants, 660.
Neurosis, value of strychnine in, 423
Nevus, croton oil in locally, 27
Night sweats, treated with atropia, 37, 47, 396; of phthisis, oxide of zinc in, 406
Nipples, sore, treatment of, 96, 499, 551, 720
Nitrate of lead, locally in onychia maligna, 304
Nitric acid in uterine diseases, 29; in hooping cough, 309
Nitrous oxide, death from use of, 103
Nitrite of amyl, uses of, 366; in epilepsy, 397; action of in the moribund state, 613
Nitrate of silver, in orchitis, 415
Nutritious injections, 663
Nux vomica, recommended in obstetrics, &c., 306; in alcoholism, 429

### O.

Oatmeal, experiments with as a diet for children, 235; value of, 425
Oil, from the peanut a substitute for olive oil, 309; value of in surgical dressings, 497
Onychia, syphilitic, acid nitrate of mercury in, 49; maligna, nitrate of lead in, 304
Oleate of mercury in ringworm, 162
Operations, surgical summary of cases by Esmarch's methcd (bloodless), 608
Ophthalmia monatorum, essay on, by J. C. Bishop, M.D., 1
Opium, poisoning by use of oxygen in, 153
Opium, habit, medicine to aid in overcoming, 348
Opium habit, treated hypodermically, by J. B. Mattison, M.D., Chester, N. J. (read before the Meigs and Mason Academy of Medicine of Middlefort, Ohio, and ordered published), 699
Oxygen in scarlet fever, 151; in opium poisoning, 153.
Orchitis, nitrate of silver in, 415, 606; tobacco poultice in, 665
Orbit, idiopathic abscess of, 639
Ovariotomy, normal, Prof. T. G. Thomas' case of, 245
Oxygenating remedies, 415

### P.

Pancreatine, uses of, 41
Pancreas, case of cancer of, by E. Cutter, M.D., 12
Pain, an index to treatment and remedies for, 105
Pains, uterine, pressure on the abdominal walls to assist, 490
Paralysis, facial, treatment of, 180; one of the probable causes of, 349; facial with marasmus, case of, 389.
Paraphymosis, treatment of, 555
Paper, green, poisonous, 350
Paracentisis thoracis in empyema, a successfvl case of, 148
Parturition, difficult, by L. B. Anderson, M.D., Va., 707.
Patent medicines, 604
Pelvic cellutitis, 112
Penis, formation of bone in, 355
Pepsin, domestic, to prepare, 171; remarks on the administration of, 371; cheap method to prepare, 498; in diptheria, 551; value of, 596; formula for use of, 556
Perchloride of iron, intra uterine injection in uterine hemorrhage, 45; administration of, 729.
Pertussis, pathognomonic sign of, 305, 555; nitric acid in, 309; treatment of, 363
Phlegmasia dolens, 484
Phosphorus, formula for giving, 30, 99; in cerebral parises, 92; in neuralgia, 166, 546; mixture of, 241, 428; in melancholia, 430; pill of, 661
Phosphorus acid, its compounds and their therapeutic value in nervous diseases, read before the Meigs County Medical Society by T. Curtis Smith, M.D., 688
Phosphide of zinc, formula to use, 425
Phosphatic food in debility, 364
Pharyngitis, nervous treatment of, 369
Phosphate of lime, 647
Phosphates, formula for giving, 169
Phthisis, treatment of, 49; importance of rest in treating, 50
Phthisis, communicability of, 648
Phthisis, cure of a case of 474
Physostigma, hypodermic use of, 328
Phytolacca decandra in mastitis, 24; essay on, by C. H. Tidd, M.D., 85; in cutaneons eruptions, 396
Pills, quinine, 42; for gout, 177.
Pitting from small pox, to prevent, 52, 407
Placenta, partial ossification of, 23
Pleuritis, treatment of, 545
Pneumonia, veratrum in, 20; acute, aconite in, 51; remarks on treatment of, 220; treatment of at the Charity Hospital, N. Y., 405; in children, veratrum in, 547
Pneumogastric nerve, physiology of, 477
Poisonous bites and stings of insects, treatment of, 88, 89
Poisoning by rhus, carbolic acid in, 306; by opium, belladonna in, 136; by rhus, treatment of, 419; value of apomorphia as an emetic in, 422; by camphor, 498
Poisonous action of analine dyes, 309
Podophyllin, Scudder on uses of, 113, 543
Post partum hemorrhage, anticipatory treatment of, 395
Potatoes, supposed bad effects of use of, 554
Potassium bromide, use of, 726
Potassium brom., of risks in using, 46
Potassium, chloride of in epilepsy, 662
Pregnancy, question of using quinine in, 40; vomiting in cured by enemata of brom. potassium, 309; persistent hymen in, 480
Propylamin, in acute rheumatism, 551
Prolapsus ani, ergotin in, hypodermically, 371, 303
Prescribing; thoughts on, by F. A. Ramsey, M.D., 191
Prostrate, senile enlargement of, 205
Prurigo and pruritus, carbolic acid internally in, 92
Pruritus vulvæ, treatment of, 170
Prurigo, treatment of, 349
Pruritus, sulpho-carbolate of zinc in, 676
Psoriasis, acetic acid in, 179, 238
Pulmonary cavities, local treatment of, 781
Puerperal amaurosis, importance as a symptom, 38
Puerperal mania, 82
Puerperal mania, chloral, brom. pot. in treatment of, 734
Puerperal insanity, 709
Puerperal convulsions, cimicifuga in, 171; bleeding in 350; hyd. chloral and morphia in, 370
Puerperal thrombosis, ammonia in, 430.
Puerperal fever, nature and treatment of, 594
Pulmonary cavities, treatment of, 655
Purgative mixture of Vienna, 661
Pyemia after amputation, treatment of, 397; turpentine in, 655; treatment of, 666
Pyelitis, new sign of, 674

### Q.

Quinine, should it be administered during pregnancy, 40
Quinine and morphia, how to distinguish each, 42
Quinine, sulphate, to make pills of, 42
Quinine, in pertussis, 111, 528
Quinine, by the rectum, 169
Quinine, pillular mass of, 175
Quinine, tannate of, 397
Quinine, suppositories by rectum, 427

### R.

Rattlesnake, its poison and antidote; essay by G. M. Rives, M.D., 505
Registration, importance of, 16
Rectum, treatment of ulceration of, 478, 558; hemorrhage from, simple plug to arrest, 357
Rectum, injection inefficient, 676
Resection of hip, fracture during, 93

Respiratory centre, 535
Retinal pulsation a symptom exophthal, mos, 346
Reviews, editorial, 739
Rheumatism, articular, acute, treatment of, 734
Rheumatism acute, treatment of, 233, 234, 487; popylamin in, 551; external application in, 406; new remedy in, 536; bromide of ammonium in, 657
Rhus, poisoning by, carbolic acid in locally, 306; treatment of, 419

S.

Salicine as an antiperiodic, essay on, by N. B. Toby, M.D., 579; as a remedy in diarrhœa and dysentery, essay on by J. C. Bishop, M.D., 585
Salivation, atropia in, 37; a case of, 468
Sarracenia purpurea, claimed as a remedy for small pox, 104
Scarlatina, formula to use belladonna as a preventive, 48; oxygen in, 151; treatment of, 411
Sciatic nerve, excision of five inches of, 229
Sciatica, actual cantery in, 182; hyd. chloral in, 370
Science abstract, commercial value of, 480
Sclerotic, melanotic sarcoma of, 36
Scybalas, mass of mistaken for ovarian tumor, 226
Sea sickness and vomiting, belladonna plaster in, 40
Secondary hemorrhage, case of, 27
Sewing machines, bad effects from constant use of, 224
Septicemia after amputation, treatment of, 397
Shingles, treatment of, 101, 553
Sick stomach, food for, 99
Silver oxide of, in menorrhagia, 179
Silicate of soda, in vesical disease, 229; dressing in fractures, 406; in gonorrhœa, 556
Singular case, 526
Sinapisms, new way to prepare, 241
Skin, diseases of, value of phytolacca in, 396
Sleep, cerebral circulation in, 150; production of anesthesia during, 535
Sleeping sickness, 348
Snuff, anti-neuralgic 50, 427
Snuff, catarrh, 658
Soap, formula for, 50
Sore nipples, treatment of, 96
Solder for broken metal, 417
Sound, in diagnosis of uterine disease, 354
Spleen, malarial enlargement of treatment, 23, 113; and kidneys found double, 358; action of cold water on when exposed in animals, 361; excision of portion of and recovery, 656
Speculum, new vaginal, 598, 678
Special senses, 522
Spinal anemia, case of, 232
Sponge tents, to make inoffensive, 229
Spermatorrhœa, formula for, 99; mental treatment of, 111
Stomatitis, ulcerative, hydrochloric acid locally in, 48, 92
Stings of bees and wasps, lime water in, 480
Sterility caused by removal of mammæ, 306
Stone, diagnosis of, 653
Stomach, gas in, 175; tube, introduction of, 176; irritable, raw beef in, 406
Strange suggestion, 676
Stricture, impassible, treatment of, 101; of cervix and perineum in labor, treatment of, 557
Strangulated hernia, tractile method of reducing, 305
Strychnia and atropia in ophthalmia, 157
Strychnia, in the neuroses, 423; as a poison, 449
Strychnia, antidotes to poisonous action of, 659
Syphilitic urethral discharges, 94
Syphilis, permanence of, 107; laws of congenital, 226
Syphilis, congenital, 228; iodoform in, 163; sources of 356
Syphilis, vegetable cure for, 362; rules for use of mercury, 525
Syphilis, treatment of, 545, 725
Surgery cosmetic, 731
Sycosis, treatment of, 110
Syrup bromide of iron, 427

T.

Tannin, antidote for strychnine, 154
Taraxacin as a remedy, 104
Tartrate of sodium, 110, 413
Taenia, remedies for, 431, 491, 165, 224
Tears, physiology of, 347
Tests for impurities in water, 731
Test for morphia, 42; for blood, 308
Testicle, non-descent of right, 392; inflamed, nitrate silver in, 415
Tetanus, treatment of, 51, calabar bean in, 329; hydrate chloral in, 362; brandy in, 224; and strychnia poisoning, differential diagnosis of, by W. P. Wells, M.D., 333
Therapeutic progress, 471
Throat, nose and ear, topical medication of, 539
Thrombosis, puerperal, ammonia in, 430
Tincture of eucalyptus globules in intermittent fever, 730
Tonsilitis epidemic, 257
Toothache, acetate of lead in, 105; anodyne for, 676
Transfusion, modes of performing, 14, 43
Tracheotomy in an infant, recovery, 658
Trachoma, 98
Trichina, 225
Tumor, encysted, of the neck, 30; of the eye, 36; elastic ligature in removal of, 94; pathogenesis of, 347
Turning in labor, 361

### U.

Ulcers, of vulva, 548; chronic, 182
Ulcers, treated by magnesia dressings, 733
Umbilical cord, care of, 7; tying of, 7
Umbilical hernia, infantile, 8
Umbilicus, papilloma of, 494
Urine, suppression of in typhoid fever, 495; incontinence of in children, 550, 556, 636; retention of from stricture, 559; retention of, 224; phosphoric acid in, in brain disease, 337, 654; aspiration of, 657
Urine, retention of, relieved by use of ice in rectum, 733
Urethra, stricture of, 481; extraction of foreign bodies from, 531
Uremia, acute, morphia in, 428
Urethro-cystitis, gelseminum in 426
Urination, painful, treatment of, 181
Urethral discharges, syphilitic, 94
Urethrotomy in the aged, 559
Uterus, ulceration of the neck of, 546; occlusion of at time of labor, 147; retroversion of, 295
Uterine injections, danger of, 662
Uterine diseases, nitric acid in, 29; hemorrhage, injection of solution of perchloride of iron in, 45; discharges, treatment of, 109; fibroids, ergotin in hypodermically, 307; sound, in diagnosis, 354; hemorrhage periodical, treated by acid nitrate of mercury, 422; tonic, 181
Uterus, treatment of retroflexions by Ely Van De Warker, M.D., 629
Uvula, functions of, 654

### V.

Vaccination, animal, 304
Vaginal discharges, bromo-chloralum in, 420
Vaginitis, acute, treatment of, 733
Valerian in diabetes, 48
Variola, to prevent pitting in, 52; origin of at Houston, Texas, 89
Varicose veins, external treatment of, 491; hyd. chloral in, hypodermically, 617; external treatment of, 301
Veratrum viride in pneumonia, 20; in cerebro-spinal meningitis, 89; in inflammation, 103
Version podalic, 351
Villate's mixture and its uses, 721
Vomiting, of pregnancy, induction of abortion for, 495; treatment of, 324, 352; enemas of bromide potassium in, 309, 500
Vomiting, from cough, 430
Vomiting, belladonna plaster in, 40
Vomiting, chronic in children, 497
Vulva, treatment of ulcers of, 163

### W.

Water, tests for impurities of, 731
Water, used hypodermically, 496; alum, to purify, 675
Weight, average, of infants at birth, 133, 465
What was it? by J. W. Booth, M.D., 133
Whooping cough, pathognomonic sign of, 305; remedy for, 363
Whiskey, hypodermic use of, 398
Witness, medical, 352
Worm remedy, 240
Wounds, powdered coal tar in, 552

### X.

Xylol, 472

### Y.

Year, the old and the new, 736.
Yellow fever, carbolic acid a disinfectant to prevent the spread of, 38

### Z.

Zona treated by collodion and morphia, 431, 662

### ASSOCIATE EDITORS:

**GEORGIA.**

James A. Long, M.D.
W. L. M. Harris, M.D.
H. L Battle, M.D.
W. C. Musgrove, M D
L. B. Bouchelle, M.D.
Thomas Burdell, M.D.
E. H. W. Hunter, M.D.
V. S Hitt, M.D.
J. E. Pope, M.D.
A. H. Brantley, M.D.
J. J. Knott, M.D.
J. C. Wisdom, M.D.
W. W. Leake, M.D.
S. G. WHITE, M.D.
W. H. HALL, M.D.
G. D. CASE, M.D.

**KENTUCKY.**

W. S. Taylor, M.D.
B. W. Stone, M.D.
Charles Kearns, M.D.

**TEXAS.**

S. M. Welch, M.D.
T. J. Heard, M.D.
W. A. Heard, M.D.

**TENNESSEE.**

J. D. Tucker, M.D.

**ALABAMA.**

J. C. Knox, M.D.
Geo. F. Taylor, M.D.
J. B. Barnett, M.D.
S. M. Hogan, M.D.
D. S. Hopping, M.D.
J. T. Searcy, M.D.
S. F. Hill, M.D.

**NORTH CAROLINA.**

J. B. Hughes, M.D.
S. S. Satchwell, M.D.
A. B. Pierce, M.D.
H. Kelly, M.D.
Geo. A. Foote, M.D.
E. A. Anderson, M.D.
H. T. Bahnson, M.D.
Willis Alston, M.D.
Thos. F. Wood, M.D.
N. J. Pittman, M.D.
John W. Booth, M.D.

**SOUTH CAROLINA.**

E. T. McSwain, M.D.
G. M. Rivers, M.D.

**OHIO.**

J. Q. A. Hudson, M.D.
C. H. Tidd, M.D.

**VIRGINIA.**

John H. Claiborne, M.D.
F. Horner, Jr., M.D.
R. J. Preston, M.D.
J. E. Lockridge, M.D.
J. S. Wharton, M.D.
Wm. O. Owen, M.D.
Sam'l P. Ripley, M.D.
Benj. Blackford, M.D.
E. A. Drewry, M.D.
Rob B. Tunstall, M.D.
W. F. Barr, M.D.
L. B. Anderson, M.D.
J. M. Lazzell, M.D.

**MISSISSIPPI.**

C. B. Gallaway, M.D.
Edward Lea, M.D.
S. V. D. Hill, M.D.
E. T. Henry, M.D.
T. P. Lockwood, M.D.
Robert Kells, M.D.

**ARKANSAS.**

A. D. Hodge, M.D.
J. K. Hodge, M.D.
A. W. Hobson, M.D.

**NEW JERSEY.**

J. B. MATTISON, M.D.

### CORRESPONDING EDITORS:

Ely Van DeWarker, M.D., New York.
C. C. F. Gay, M.D., New York, *Surgeon of Buffalo General Hospital.*
M. H. Henry, M.D., New York, *Surgeon to N. Y. Dispensary, Dep't of Venerial and Skin Diseases.*
Benjamin Howard, M.D., New York.
James Tyson, M.D., Philadelphia, Pa.
W. W. Keen, M.D. Philadelphia.
Robert Robson, M.D., Indiana.
A. Patton, M.D., Indiana.
John H. Weir, M.D., Illinois.
Thomas J Gallaher, M.D, Pennsylvania.
S. C. Busey, M.D., Washington, D. C.
Wm. R. Whitehead, M.D., Colorado Territory.

☞ The RECORD will be mailed between the 20th and 25th of each month.

